# Paradoxical Association Between Relative Cerebral Blood Volume Dynamics Following Chemoradiation and Increased Progression-Free Survival in Newly Diagnosed IDH Wild-Type MGMT Promoter Methylated Glioblastoma With Measurable Disease

**DOI:** 10.3389/fonc.2022.849993

**Published:** 2022-03-08

**Authors:** Jodi Goldman, Akifumi Hagiwara, Jingwen Yao, Catalina Raymond, Christian Ong, Rojin Bakhti, Elizabeth Kwon, Maguy Farhat, Carlo Torres, Lily G. Erickson, Brandon J. Curl, Maggie Lee, Whitney B. Pope, Noriko Salamon, Phioanh L. Nghiemphu, Matthew Ji, Blaine S. Eldred, Linda M. Liau, Albert Lai, Timothy F. Cloughesy, Caroline Chung, Benjamin M. Ellingson

**Affiliations:** ^1^ UCLA Brain Tumor Imaging Laboratory, Center for Computer Vision and Imaging Biomarkers, Department of Radiological Sciences, David Geffen School of Medicine, University of California, Los Angeles, Los Angeles, CA, United States; ^2^ Department of Radiological Sciences, David Geffen School of Medicine, University of California, Los Angeles, Los Angeles, CA, United States; ^3^ Department of Radiation Oncology, The University of Texas MD Anderson Cancer Center, Houston, TX, United States; ^4^ Department of Neurology, David Geffen School of Medicine, University of California, Los Angeles, Los Angeles, CA, United States; ^5^ Department of Neurosurgery, David Geffen School of Medicine, University of California, Los Angeles, Los Angeles, CA, United States; ^6^ Department of Psychiatry and Biobehavioral Sciences, David Geffen School of Medicine, University of California, Los Angeles, Los Angeles, CA, United States

**Keywords:** glioblastoma, dynamic susceptibility contrast perfusion MRI, MGMT methylation, rCBV, chemoradiation

## Abstract

**Background and Purpose:**

While relative cerebral blood volume (rCBV) may be diagnostic and prognostic for survival in glioblastoma (GBM), changes in rCBV during chemoradiation in the subset of newly diagnosed GBM with subtotal resection and the impact of MGMT promoter methylation status on survival have not been explored. This study aimed to investigate the association between rCBV response, MGMT methylation status, and progression-free (PFS) and overall survival (OS) in newly diagnosed GBM with measurable enhancing lesions.

**Methods:**

1,153 newly diagnosed IDH wild-type GBM patients were screened and 53 patients (4.6%) had measurable post-surgical tumor (>1mL). rCBV was measured before and after patients underwent chemoradiation. Patients with a decrease in rCBV >10% were considered rCBV Responders, while patients with an increase or a decrease in rCBV <10% were considered rCBV Non-Responders. The association between change in enhancing tumor volume, change in rCBV, MGMT promotor methylation status, and PFS or OS were explored.

**Results:**

A decrease in tumor volume following chemoradiation trended towards longer OS (p=0.12; median OS=26.8 vs. 16.3 months). Paradoxically, rCBV Non-Responders had a significantly improved PFS compared to Responders (p=0.047; median PFS=9.6 vs. 7.2 months). MGMT methylated rCBV Non-Responders exhibited a significantly longer PFS compared to MGMT unmethylated rCBV Non-Responders (p<0.001; median PFS=0.5 vs. 7.1 months), and MGMT methylated rCBV Non-Responders trended towards longer PFS compared to methylated rCBV Responders (p=0.089; median PFS=20.5 vs. 13.8 months).

**Conclusions:**

This preliminary report demonstrates that in newly diagnosed IDH wild-type GBM with measurable enhancing disease after surgery (5% of patients), an enigmatic non-response in rCBV was associated with longer PFS, particularly in MGMT methylated patients.

## Introduction

Glioblastoma (GBM) is the most aggressive and treatment resilient primary brain malignancy amongst adults (1, 2). The current standard of care for GBM is maximal safe resection of the tumor followed by radiation therapy (RT) and concomitant temozolomide (TMZ) (3) with or without tumor treating fields (4). A pathological hallmark of GBM is the formation of new blood vessels, or angiogenesis. Angiogenesis is critical for transformation from lower to higher grade gliomas, and studies have shown there is strong association between angiogenesis and degree of malignancy and tumor growth rates (5–8). Thus, peritumoral hemodynamics might provide important biological information to better understanding early changes in the tumor microenvironment as a result of standard chemoradiation.

Dynamic susceptibility contrast (DSC) perfusion MRI is an imaging technique sensitive to tumor vascularity that aids in diagnosis, treatment monitoring, and survival prediction (9, 10). One particularly useful hemodynamic measurement is the relative cerebral blood volume (rCBV), which is defined as the volume of blood for a given mass of brain tissue relative to a tissue of reference, often contralateral white matter. Many studies have shown that rCBV from DSC is useful in differentiating between pseudoprogression and tumor progression in high-grade glioma after chemoradiotherapy (CRT), with lower rCBV suggesting possible pseudoprogression and longer survival compared with true progression (11–17). Furthermore, some studies have suggested that a decrease in rCBV of high-grade glioma following CRT is predictive of improved OS (18, 19). However, these reports involved a very small number of patients, included both IDH mutant and wild type GBM, included a mixture of gross total and subtotal resection patients, and did not incorporate the assessment of MGMT promoter methylation status, an epigenetic marker known to be strongly associated with increased survival for patients with GBM treated with chemoradiation (20). Hence, we hypothesized that patients with newly diagnosed, IDH wild-type, MGMT promoter methylated GBM that demonstrate an interval decrease in rCBV following CRT would experience the greatest survival benefit. Therefore, the aim of the current study was to determine the association between the rCBV response and the survival of patients with GBM, when MGMT promotor methylation status is considered.

## Materials and Methods

### Patient Characteristics

A total of 1,153 newly diagnosed IDH wild-type GBM patients with available perfusion MRI and clinical information were screened at the University of California Los Angeles (UCLA) and MD Anderson Cancer Center (MDA) under IRB approved protocols. Following diagnosis, all patients underwent surgical resection followed by concomitant TMZ and RT followed by adjuvant TMZ until progression. All patients were also required to have multiparametric MRI including DSC perfusion sequence before and after RT; the pre-RT MRI was no earlier than seven weeks before starting treatment (median, 11 days; range, 0–46) and the post-RT MRI was no later than eight months following the completion (median, 27 days; range, 0–234). Patients were excluded from the study if the post-surgical enhancing disease before CRT was less than 1 mL measured on post-contrast T1-weighted imaging, constituting subtotal resection and approximate “measurable” disease (e.g. 1cm x 1cm) according to the Response Assessment in Neuro Oncology (RANO) criteria (21). Patients with tumors in anatomical locations susceptible to susceptibility-induced geometric distortions, such as near the skull base, were also excluded. Out of 1,153 patients screened, only 53 patients (4.6%) had measurable enhancing disease (>1mL) prior to CRT, with N=40 patients from UCLA and 13 patients from MDA. These 53 patients had a median age of 63 years (range 20–74 years) at the time of diagnosis and were divided relatively evenly by sex (29 males, 24 females). Forty-seven out of the 53 total patients (88.7%) had MGMT promotor methylation status available. Patient and group characteristics are outlined in **Table 1**.

**Table 1:** Descriptive statistics and associations between rCBV Responders and rCBV Non-Responders.

rCBV Responder and Non-Responder Groupings				
	All Patients (n = 53)	rCBV Responders (n = 24)	rCBV Non-Responders (n = 29)	p-value*
Age, median (range), year	63 (20-74)	66 (27-74)	61 (20-72)	
Gender, male (%)	29 (54.7)	15 (62.5)	14 (48.2)	
Methylation Status				
Methylated (%)	19 (35.8)	8 (33.3)	11 (37.9)	
Unmethylated (%)	28 (52.8)	14 (58.3)	14 (48.3)	
Unknown (%)	6 (11.3)	2 (8.3)	4 (13.8)	
rCBV, median (range)				
Pre-CRT	1.42 (0.03-5.87)	2.20 (0.09-5.87)	0.81 (0.03-5.52)	p<0.001
Post-CRT	1.24 (0.00-7.75)	1.22 (0.00-4.74)	1.26 (0.29-7.75)	p=0.144
Tumor Volume, median (range), mL				
Pre-CRT	8.41 (1.07-42.64)	5.96 (1.07-42.64)	10.37 (2.34-40.78)	p=0.126
Post-CRT	11.59 (0.57-56.80)	10.68 (0.57-40.863)	11.69 (1.25-56.80)	p=0.412

rCBV, relative cerebral blood volume; CRT, chemoradiation therapy.

*P-values compare rCBV Responders vs rCBV Non-Responders using two-tailed T-test.

### MRI Acquisition

MRI was acquired using 1.5-T or 3-T scanners. The DSC perfusion MRI was performed with an administration of 0.1 mmol/kg dose of gadopentate dimeglumine (Gd-DTPA; Magnevist, Bayer Schering Pharma, Leverkusen,Germany), 0.025 mmol/kg for preload dosage to mitigate T_1_-based leakage contamination and the remaining 0.075 mmol/kg for dynamic bolus administration. The wait time between the preload dose and the onset of baseline imaging was 2 minutes. The sequence is composed of a series of gradient-echo EPI acquisitions, with TE/TR = 23-35/1500–2000 ms, flip angle = 60, field-of-view, 240 × 240 mm, matrix size = 128 × 128, slice thickness = 4–5 mm with an interslice gap of 0–1.5 mm, number of baseline acquisitions before contrast agent injection = 10–25, and number of timepoints = 120 at 1.5 T; TE/TR = 17–45/1120–2550 ms, flip angle = 60 or 90, field-of-view, 240 × 240 mm, matrix size = 128 × 128, slice thickness = 4–5 mm with an interslice gap of 0–2 mm, number of baseline acquisitions before contrast agent injection = 10–25, and number of timepoints = 40–120 at 3 T.

In addition to DSC-perfusion MRI, all patients received the anatomic images including T_2_-weighted fluid attenuated inversion recovery (FLAIR) images, T_2_-weighted turbo spin-echo images, and parameter matched, T_1_-weighted scans before and following injection of contrast according to the standardized brain tumor imaging protocol (22).

### Post-Processing

The DSC-MRI data was first motion corrected using FSL (*mcflirt*, FMRIB software library, Oxford, England). For post-processing and the creation of rCBV maps, bidirectional contrast agent leakage was performed for all subjects using MATLAB (MathWorks, Natick, Massachusetts) (23). The rCBV maps were rigidly registered to post-contrast T_1_-weighted images for subsequent analysis. Regions of contrast enhancement were contoured on post-contrast T_1_-weighted images using Analysis of Functional NeuroImages (AFNI) software (NIMH Scientific and Statistical Computing Core; Bethesda, MD, USA) for 40 patients at UCLA, and Raystation 10B DTK was used in addition to AFNI for contouring 13 patients at MDA. Tumor regions of interest (ROIs) were semi-automatically created, by first encircling the entire contrast-enhancing lesion on continuous slices. Next, a threshold was set to capture the contrast enhancing region. Tumor volumes were calculated from the ROI as a volumetric assessment for each pre- and post-CRT scan. ROIs of normal appearing white matter (NAWM) were contoured on the brain contralateral to the tumor on post-contrast T_1_-weighted images. Normalized rCBV values were determined by dividing the median rCBV within the tumor region by the median rCBV of the NAWM ROI. The change in rCBV following CRT was calculated using the following equation:


$$\text{rCBV}=\left[\frac{post-CRT\;tumor\;rCBV}{post-CRT\;NAWM\;rCBV}\right]-\left[\frac{pre-CRT\;tumor\;rCBV}{pre-CRT\;NAWM\;rCBV}\right]$$

At UCLA, a team of trained lab technologists created initial ROIs and all final ROIs were reviewed by a neuroradiologist (A.H.) with 10 years of experience in neuroimaging analysis. At MDA, a team of trained lab technologists created initial ROIs. All final ROIs were reviewed by a radiology resident (M.F.) and more complicated cases were reviewed by a neuro-radiation oncologist (C.C.) with over 10 years of experience in neuroimaging analysis.

### rCBV Responders and Non-Responders

**Figure 1:**
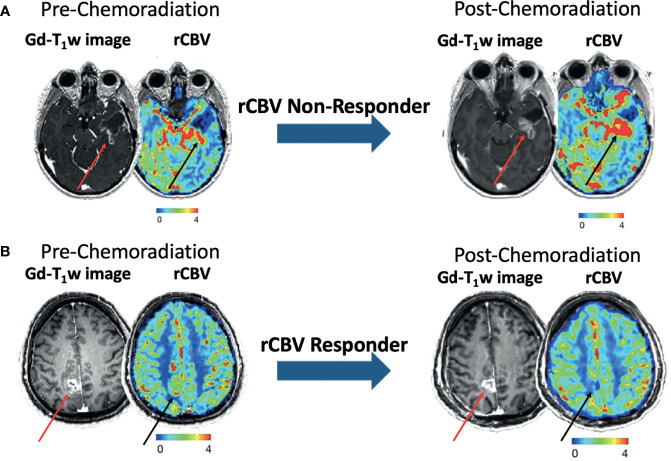
Examples of pre- and post-CRT (left and right) post-contrast T_1_-weighted images and rCBV maps from patients categorized as rCBV Non-Responder **(A)** and rCBV Responder **(B)**. Red and black arrows correspond to the ROIs on the T_1_-weighted images and rCBV maps, respectively.

Patients were further divided into two groups: rCBV Responders and Non-Responders. rCBV Responders were defined as patients that demonstrated at least a 10% decrease in rCBV following CRT (**Figure 1B**), where rCBV Non-Responders were defined as patients that demonstrated either an increase or less than a 10% decrease in rCBV following CRT (**Figure 1A**). At least a ten percent decrease was deemed necessary to define rCBV Responders to ensure a clinically relevant decrease was observed, because the normal variation of rCBV in high grade tumor is known to be approximately 10% (24, 25).

### Statistical Analysis

Kaplan-Meier estimation followed by Gehan-Breslow-Wilcoxon (GBW) testing was performed to investigate the association between change in enhancing tumor volume, change in rCBV, or MGMT promotor methylation status, and progression-free survival (PFS) or overall survival (OS) using GraphPad Prism (version 8.4.3, GraphPad Software, San Diego, California, USA). Tumor progression was determined by neuro-oncologists at each institution using RANO criteria (21). GBW testing was used to put more weight on early time points given that the cohort is relatively small. We also performed conventional log-rank analysis. A p-value < 0.05 was considered statistically significant. OS and PFS were defined as time from the end of RT to death and progression, respectively, or censor date.

## Results

Twenty-four patients were classified as rCBV Responders (45.3%) and 29 patients were classified as rCBV Non-Responders according to this convention. The median OS for all patients was 16.3 months and the median PFS for all patients was 7.8 months. At the time of analysis, 30 patients (57%) died and 23 patients (43%) were still alive. Of the rCBV Non-Responders, 17 patients (59%) died and 12 patients (41%) were still alive. Of the rCBV Responders, 13 patients (54%) died and 11 patients (46%) were still alive. At the time of analysis, 43 patients (81%) experienced disease progression while 10 patients (19%) did not. Of the rCBV Non-Responders, 22 patients (76%) experienced disease progression while 7 patients (24%) did not. Of the rCBV Responders, 21 patients (88%) experienced disease progression while 3 patients (13%) did not.

**Figure 2:**
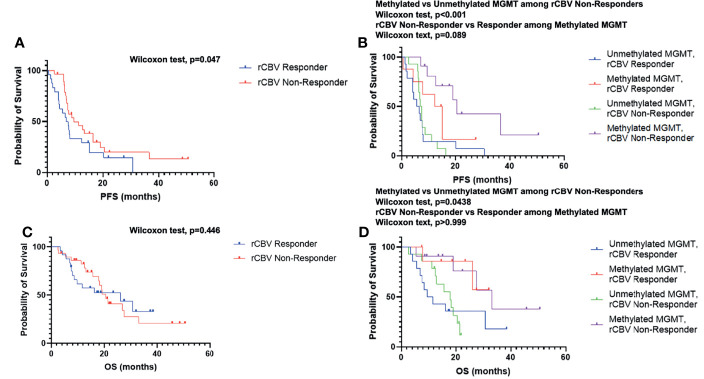
Kaplan–Meier survival curves of rCBV Responder and rCBV Non-Responder for PFS **(A)** and OS **(C)**. Kaplan–Meier survival curves of rCBV Responder and rCBV Non-Responder stratified by MGMT methylation status for PFS **(B)** and OS **(D)**.

**Figure 3:**
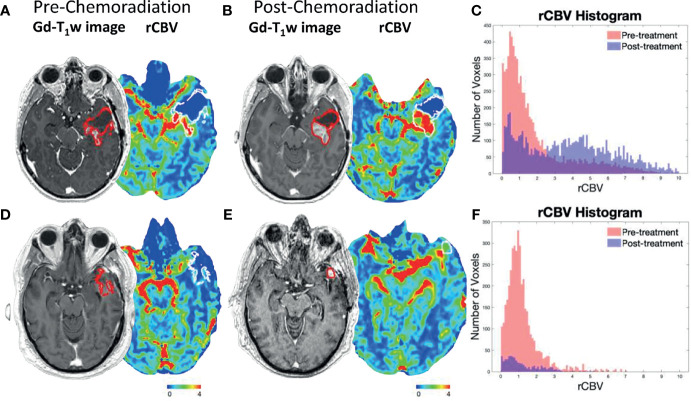
Representative post-contrast T1-weighted images and rCBV maps for a rCBV Non-Responder with Methylated MGMT **(A, B)** and rCBV Responder with Methylated MGMT **(D, E)**. Tumor ROIs are outlined in red on post-contrast T1-weighted images and in white on rCBV maps. The **(A)** pre- and **(B)** post-CRT scans of a rCBV Non-Responder with Methylated MGMT who experienced PFS of 36.6 months and OS of 45.7 months. A rCBV histogram demonstrates an increase in rCBV values from pre- to post-CRT **(C)**. The **(D)** pre- and **(E)** post-CRT scans of a rCBV Responder with Methylated MGMT who experienced PFS of 15.2 months and OS of 26.1 months. rCBV histogram demonstrates a decrease in rCBV values from pre- to post-CRT **(F)**.

When comparing rCBV Non-Responders and rCBV Responders, rCBV Non-Responders experienced an *improved* PFS (GBW: p=0.047; log-rank: p=0.11; median PFS: 9.6 vs. 7.2 months; **Figure 2A**), which directly contradicted our original hypothesis. There was no significant difference when comparing OS between the two groups (GBW: p=0.45; log-rank: p=0.93; median OS: 20.6 vs. 26.1 months; **Figure 2C**). Patients were further subdivided into four groups: rCBV Responder with Methylated MGMT (n=8), rCBV Responder with Unmethylated MGMT (n=14), rCBV Non-Responder with Methylated MGMT (n=11), and rCBV Non-Responder with Unmethylated MGMT (n=14). In patients with MGMT methylation status available (N=47), rCBV Non-Responders with Methylated MGMT showed improved PFS compared to rCBV Non-Responders with Unmethylated MGMT (GBW: p<0.001; log-rank: p=0.0004; median PFS: 20.5 vs. 7.1 months; **Figure 2B**). rCBV Non-Responders with Methylated MGMT also had improved PFS when compared to rCBV Responders with Methylated MGMT, although not significant (GBW: p=0.089; log-rank: p=0.11; median PFS: 20.5 vs. 13.8 months; **Figure 2B**). Representative pre- and post-CRT T1 post contrast and rCBV maps for a rCBV Non-Responder with Methylated MGMT and a rCBV Responder with Methylated MGMT are shown in **Figure 3**. rCBV Non-Responders with Methylated MGMT had improved OS compared to rCBV Non-Responders with Unmethylated MGMT (GBW: p=0.044; log-rank: p=0.015; median OS: 33.1 vs. 17.9; **Figure 2D**). However, there was no significant OS difference between rCBV Non-Responders with Methylated MGMT and rCBV Responders with Methylated MGMT (GBW: p>0.999; log-rank: p=0.996; median OS: 33.1 vs. 21.01 months; **Figure 2D**). Between rCBV Non-Responders and rCBV Responders with Unmethylated MGMT, there were no significant differences found for PFS or OS (PFS: GBW p=0.23, log-rank p=0.78, 7.15 vs 6.05 months, **Figure 2B**; OS: GBW p=0.26, log-rank p=0.71, 17.94 vs 10.69 months, **Figure 2D**). Regardless of rCBV-Response status, patients with Methylated MGMT experienced improved PFS and OS (PFS: GBW p=0.0003, log-rank p<0.0001, 15.21 vs 6.75 months, **Figure 2B**; OS: GBW p=0.0038, log-rank p=0.0018, 33.05 vs 15.7 months, **Figure 2D**).

**Figure 4:**
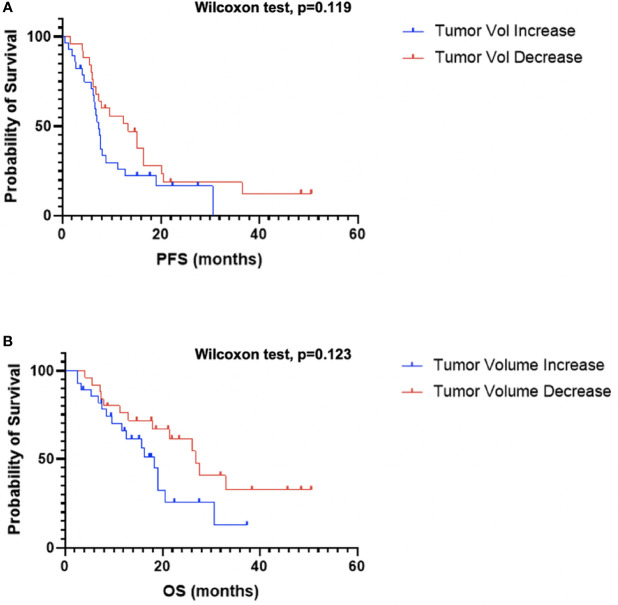
Kaplan–Meier survival curves of tumor volume change PFS **(A)** and OS **(B)**.

Next, as a quality control step, the impact of change in tumor volume on survival was examined as a decrease in tumor volume has been consistently reported to be associated with improved OS. Patients were divided into two groups depending on if tumor volume increased (n=28) or decreased (n=25) following CRT. Patients that demonstrated a decrease in tumor volume following CRT generally experienced an improved OS compared to patients with an increase in tumor volume, although not statistically significant (GBW: p=0.12; log-rank: 0.076; median OS: 26.8 vs. 18.3 months; **Figure 4B**). There was no significant difference when comparing PFS between the two groups (GBW: p=0.12; log-rank: p=0.15; median PFS: 13.4 vs. 7.5 months; **Figure 4A**).

## Discussion

In direct conflict with our original hypotheses and conventional wisdom, our results demonstrate that IDH wild-type MGMT methylated rCBV Non-Responders exhibiting either had stable or increasing rCBV following CRT experienced significantly *improved* PFS. Even though previous studies have suggested a decrease in rCBV in high-grade glioma following CRT should be associated with longer PFS and OS, the current study did not confirm these results. In a study by Mangla et al. from 2010 (18), often cited as supporting the association between change in rCBV and PFS or OS, investigators found that the percentage change in rCBV and absolute measures of rCBV, along with extent of resection, were associated with OS. However, this study was relatively small, involved patients with very small enhancing tumors after surgery, IDH and MGMT status were not considered, only 2D measurements of tumor size and perfusion were considered (instead of the entire volumetric region of interest), and a non-traditional approach to contrast leakage correction was used, it is difficult to delineate the influence of these factors on their results and interpretation. Interestingly, a previous study by Larsson et al. from 2020 did not show a significant association between continuous perfusion parameters and survival following CRT (19). Instead, this study showed that a combination of change in K^trans^ from dynamic contrast enhanced (DCE) perfusion MRI and change in rCBV within the T2 hyperintense lesion (not the enhancing lesion) together were prognostic for OS (19). Additionally, many of these patients had small “non-measurable” lesions, with only 9 of 23 patients having subtotal resection and a measurable target lesion, and again IDH and MGMT status were not taken into consideration.

**Figure 5:**
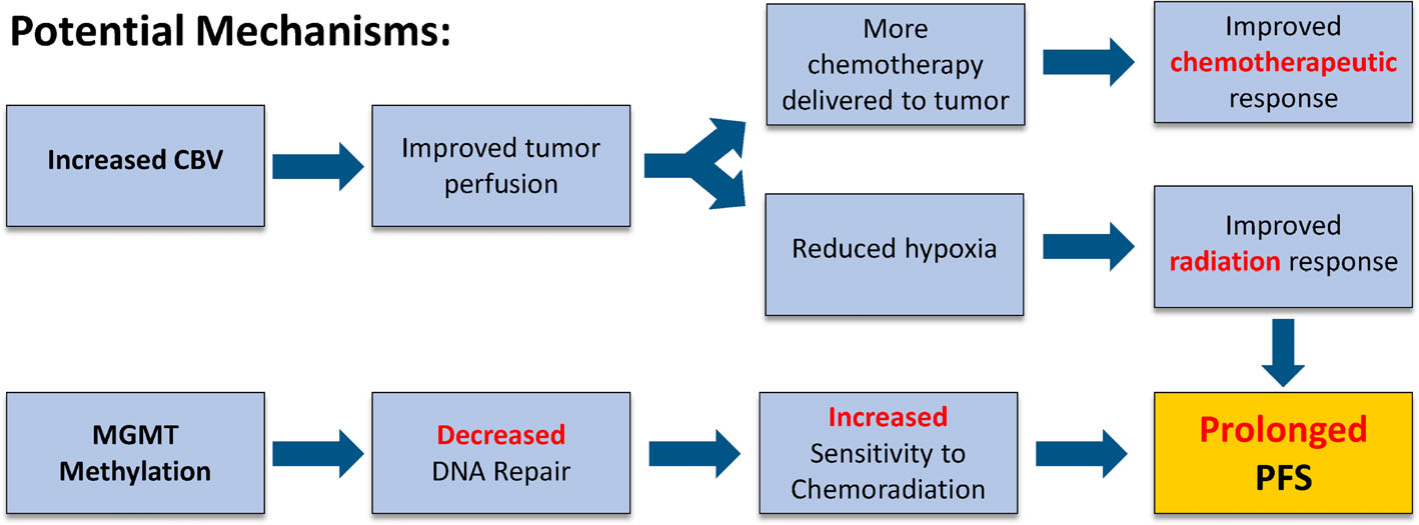
Potential mechanisms to explain PFS benefit of rCBV Non-Responder with Methylated MGMT.

In the current study, we found that newly diagnosed IDH wild-type, MGMT promoter methylated GBM exhibiting an increase in rCBV demonstrated the greatest survival benefit, particularly in terms of PFS. There are two potential and possible synergistic explanations for this observation (**Figure 5**). An increase in rCBV following CRT (i.e. rCBV Non-Responders) suggests that tumor perfusion may be improved, providing a higher concentration of chemotherapy within the tumor, improving chemotherapeutic response (26). Additionally, if more blood is delivered to the tumor it may also have a higher degree of oxygenation, leading to improved radiation response (27). Prior translational research using a U87 rat model demonstrated that treatment with an antiangiogenic therapy (Cediranib) alongside traditional chemotherapeutics increased the concentration of TMZ in the tumor bed (28). Batchelor et al. (26) found that patients with GBM treated with chemoradiation plus Cediranib demonstrated an increase in perfusion and experienced significantly improved survival compared with patients treated with CRT alone. This effect may be due to the antiangiogenic therapy normalizing blood flow and enhancing drug delivery (26). These effects are potentially intensified in patients with MGMT promoter methylation, for which therapeutic benefits from alkylating agents are well documented. In this way, increased rCBV and MGMT methylation during CRT treatment may work together to provide the greatest therapeutic benefit to IDH wild-type GBM patients.

There were a few limitations in and caveats to the current study that should be addressed. First, the sample size was relatively small leading to potential bias in our results. This was particularly evident when patients were divided into four groups based on rCBV response and MGMT methylation status, and only eight patients were categorized as rCBV Responder with Methylated MGMT. Given that more than 1,000 patients were screened and we found less than 50 patients meeting our inclusion and exclusion criteria, the observed phenomena only really apply to a subset of patients with (1) “measurable” enhancing tumor after surgery and after completion of CRT (i.e. subtotal resection), and (2) those with tumors in regions of the brain less susceptible to susceptibility-induced geometric distortions (e.g. not tumors near the skull base, auditory canals, or the orbitofrontal region of the prefrontal cortex). Second, DSC perfusion imaging protocols were not fully standardized and both 1.5 and 3 T scanners were used in this study. Tradeoffs exist between high and low TR/TE/flip angle and many differing acquisition strategies are used throughout the literature (29). The current guideline states that a full-dose preload provides a better accuracy than a fractional preload that was used in this study (29). Theoretically, perfusion measurement is independent of field strength, even though a higher field strength may benefit from a higher contrast-to-noise ratio (30). Third, due to the small sample size, we could not reliably perform survival analysis using age and sex as covariates. Additionally, since this was a retrospective study, timing between treatments and imaging, chemotherapy combinations, and steroid administration was not standardized amongst patients, which could have had an impact on the measured perfusion response. The usage of steroids may decrease rCBV, but we showed that a decrease in rCBV is detrimental to PFS. Hence, our observation would not have been affected by the usage of steroids. Similarly, some patients had pre and post scans on MRI scanners of different field strengths, which might have also influenced interpretation. Also, some patients did not have post-CRT scans close to the date of completion of the treatment. There is a possibility that rCBV has been affected by the variability in timing of the post-treatment scan.

In conclusion, the results of the current study indicate that a paradoxical *non-decrease* in rCBV following CRT appears prognostic for *improved* PFS for patients with newly diagnosed IDH wild-type GBM, particularly in patients exhibiting MGMT promoter methylation. Even though this can be observed only in a small subset of patients, with only 53 out of 1153 patients eligible in this study, the information provided by this study suggests that a decrease in rCBV is not ubiquitously associated with a therapeutic or clinical benefit in patients with GBM, but instead may depend highly on genetic/epigenetic/molecular characteristics of the tumor and the specific therapies involved. This type of information is important for understanding the utility and limitations of DSC perfusion MRI as a tool for personalized patient care in GBM.

## Data Availability Statement

Datasets analyzed during this study are available from the corresponding author on reasonable request.

## Ethics Statement

The studies involving human participants were reviewed and approved by IRB Committee at University of California, Los Angeles Health and MD Anderson Cancer Center. Written informed consent for participation was not required for this study in accordance with the national legislation and the institutional requirements.

## Author Contributions

JG: designing the study, analysing the data, and preparing the manuscript. AH: preparing the manuscript. CT: analysing the data. JY, CR, CO, RB, EK, MF, CT, LE, BC, ML, WP, NS, PN, MJ, BSE, LL, AL, TC, CC, and BME: designing the study and providing expert opinion. All the authors have contributed in designing and conduct of study, analysis of results and preparation of manuscript. All authors contributed to the article and approved the submitted version.

## Funding

This work was supported by American Cancer Society (ACS) Research Scholar Grant (RSG-15-003-01-CCE) (BME), the UCLA SPORE in Brain Cancer (NIH/NCI 1P50CA211015), and in part by Cancer Center Support Grant P30 CA016672 from the National Cancer Institute of the National Institutes of Health, to The University of Texas MD Anderson Cancer Center.

## Conflict of Interest

BME is a paid consultant and an advisor for Medicenna, MedQIA, Servier Pharmaceuticals, Siemens, Janssen, Imaging Endpionts, Kazia Pharmaceuticals, Chimerix, Sumitomo Dainippon Pharma Oncology, ImmunoGenesis, Ellipses Pharma, Monteris, Global Coalition for Adaptive Research, Neosoma, and Alpheus Medical. CC has research funding from Siemens Healthineers and RaySearch Laboratories.

The remaining authors declare that the research was conducted in the absence of any commercial or financial relationships that could be construed as a potential conflict of interest.

The handling Editor declared a past co-authorship with one of the authors BME.

## Publisher’s Note

All claims expressed in this article are solely those of the authors and do not necessarily represent those of their affiliated organizations, or those of the publisher, the editors and the reviewers. Any product that may be evaluated in this article, or claim that may be made by its manufacturer, is not guaranteed or endorsed by the publisher.
